# Medication Possession Ratio Associated with Short-Term Virologic Response in Individuals Initiating Antiretroviral Therapy in Namibia

**DOI:** 10.1371/journal.pone.0056307

**Published:** 2013-02-28

**Authors:** Steven Y. Hong, Logan Jerger, Anna Jonas, Alfons Badi, Steven Cohen, Jean B. Nachega, Jean-Jacques Parienti, Alice M. Tang, Christine Wanke, Norma Terrin, Dawn Pereko, Abraham Blom, Andrew B. Trotter, Michael R. Jordan

**Affiliations:** 1 Division of Geographic Medicine and Infectious Diseases, Tufts Medical Center, Boston, Massachusetts, United States of America; 2 Department of Public Health and Community Medicine, Tufts University School of Medicine, Boston, Massachusetts, United States of America; 3 Directorate of Special Programmes, Republic of Namibia Ministry of Health and Social Services, Windhoek, Namibia; 4 Center for Global Health, Johns Hopkins University, Baltimore, Maryland, United States of America; 5 Centre for Infectious Diseases, Stellenbosch University Faculty of Health Sciences, Cape Town, South Africa; 6 Department of Biostatistics and Clinical Research, University Hospital Center, Caen, France; 7 Institute for Clinical Research and Health Policy Studies, Tufts Medical Center, Boston, Massachusetts, United States of America; 8 Strengthening Health Outcomes through the Private Sector, Abt Associates Incorporated, Windhoek, Namibia; University of Ottawa, Canada

## Abstract

The visual-analogue scale (VAS), Likert item (rating scale), pills identification test (PIT), and medication possession ratio (MPR) provide estimates of antiretroviral therapy (ART) adherence which correlate with HIV viral suppression. These simple adherence measures are inexpensive and easy to administer; however, require validation and adjustment prior to implementation. The objective of this study was to define the optimal adherence assessment measure in Namibia to identify patients at risk for sub-optimal adherence and poor virologic response 6 months after ART initiation. We conducted a cross-sectional survey in HIV-infected adults receiving ART for 6–12 months prior to the adherence assessment. Adherence measures included 30-day VAS, 30-day Likert item, self-reported treatment interruptions, PIT, and MPR. Association of adherence measures with 6-month HIV-1 RNA level was assessed using two thresholds (1000 copies/mL and 5000 copies/mL). Adherence was assessed in 236 patients, mean age 37.3 years, 54% female. Mean adherence was 98.1% by 30-day VAS, 84.7% by 30-day Likert item, 97.0% by self-reported treatment interruptions, 90.6% by PIT, and 98.8% by MPR. Agreement between adherence measures was poor using kappa statistic. 76% had HIV-1 RNA <1000 copies/ml, and 88% had HIV-1 RNA <5000 copies/ml. MPR (continuous) was associated with viral suppression <5000 copies/ml (p = 0.036). MPR <75% was associated with virologic failure at ≥5000 copies/ml with OR 3.89 (1.24, 12.21), p = 0.013. Adherence was high with all measures. Only MPR, was associated with short-term virologic response, suggesting its cross-culturally utility for early identification of patients at high risk for virologic failure.

## Introduction

As of December 2011, over 8 million people infected with HIV were receiving antiretroviral therapy (ART) in low- and middle-income countries which represents a 26-fold increase since 2003 [1]. In June 2010, the United Nations General Assembly Special Session (UNGASS) set a goal of placing 15 million individuals on ART by 2015 [2]. Sustaining successful ART scale-up in resource-limited settings depends largely upon the ability of ART programs to deliver ART in a way that supports optimal patient adherence, thereby maximizing durability of first- and second-line regimens. Adherence to ART is a predictor of virologic suppression [3]–[8], emergence of HIV drug resistance [9]–[10], disease progression [11], and death [12]–[14].

Assessment of adherence by health care providers often results in an overestimation of patients’ medication adherence [15]. Adherence as measured by a 30-day visual-analogue scale (VAS) [16]–[17], Likert item (rating scale), pills identification test (PIT) [18], and medication possession ratio (MPR) [6], [19]–[21] have been shown to be associated with viral suppression (adherence measures defined in Methods Section). These simple adherence measures are inexpensive and easy to administer. Reliable and simple measures of adherence are essential components of ART programs, especially in resource-limited settings. Although the VAS, Likert item, PIT, and MPR have been demonstrated to be valid measures of adherence, they require validation and adjustment prior to implementation to account for local cultural and linguistic factors.

Namibia is a resource-limited country in sub-Saharan Africa that has been severely affected by the HIV epidemic. In Namibia, there are approximately 200,000 people living with HIV in a population of 2.1 million [22]–[23]. Among 15–49 year olds, approximately 18.8% are infected with HIV-1 [22]. The epidemic is predominantly spread via heterosexual contact, and prevalence estimates vary by region with up to 36% infected with HIV-1 in the most heavily-affected areas in the north [22]. ART has been available in Namibia’s private sector since 1998 and in the public sector since 2003. In the public sector, ART is provided free of charge following a population-based model of care [24]. At 90%, Namibia has one of the highest ART coverage rates in Sub-Saharan Africa with 88,717 eligible patients on ART as of December 2010 [25]. At present, ART is available at all 40 public hospitals and at an additional 111 satellite/outreach service points, as well as 30 Integrated Management of Adolescent and Adult Illness (IMAI) modules sites [24].

It is unclear which adherence measure would be most appropriate for use in Namibia’s ART program. Therefore, identifying the best tool to estimate patient adherence for Namibia would be valuable for quality-improvement of the ART program to minimize preventable HIV drug resistance and optimize patient care. The objective of this study was to define the optimal cross-cultural tool to provide ART care providers in Namibia with information to identify patients at risk for sub-optimal adherence and poor short-term virologic response.

## Methods

We conducted a cross-sectional survey in 236 HIV-infected adults in Namibia who had initiated ART 6–12 months prior to the adherence assessment. Adherence measures included a 30-day VAS, 30-day Likert item, self-reported treatment interruptions, and PIT. Pharmacy dispensing records were used to calculate MPR. A viral load was conducted 6 months after ART initiation on all ART patients and used to assess association with adherence measures. Viral load testing was conducted by the Namibian Institute of Pathology utilizing COBAS AmpliPrep/COBAS TaqMan HIV-1 Test (Hoffman-La Roche). The survey was performed between September 2010 and April 2011.

### Ethics Statement

Ethical approval was obtained from the institutional review board at Tufts University School of Medicine in Boston, USA and the Republic of Namibia Ministry of Health and Social Services Ethics and Research Committee in Windhoek, Namibia. Written informed consent was obtained from all participants.

### Study Population

The study population consisted of patients diagnosed with HIV-1 who were being prescribed adult ART and being managed at a public ART delivery site in Windhoek, Namibia. Windhoek, the largest urban setting in the country, has an estimated population of 342,000 (2009) [23].

Patients were recruited from the three major public ART delivery sites in Windhoek: Katutura State Hospital, Katutura Health Centre, and Windhoek Central Hospital. Patients were eligible to be included in the survey if they were HIV positive, started an adult ART regimen for the first time 6–12 months prior to the date of the adherence assessment, were receiving an adult ART regimen at the time of the adherence assessment, and had a routine 6-month viral load available for analysis.

### Study Adherence Measures

Adherence was measured at the routine ART clinic visit with five different adherence measures: 1) VAS, 2) 5-choice Likert item, 3) self-reported treatment interruptions, 4) PIT, and 5) MPR. The VAS asked the patient to mark an “X” on a continuous scale describing his/her level of adherence to all their antiretroviral (ARV) medications prescribed over the previous 30 days on a scale of 0% to 100%. The position indicated by the patient was converted to a percentage. The 5-choice Likert item asked the patient to choose the word that most accurately described how well they took their ARV medication during the past 30 days: “excellent”, “very good”, “good”, “fair”, and “poor”. Self-reported treatment interruptions were assessed by asking, “How many times did you ever interrupt your ART for 2 days or more.” All self-reported adherence questions were preceded by a statement asking for truthful answers, as they would be kept strictly confidential and would in no way affect their future health care. The PIT measured whether a patient was able to identify all the medication that he/she was taking and state the number of pills that should be taken and the time. The PIT used in this study was adapted from the original PIT developed by Parienti et al [18]. All routine ARV pills available in Namibia were included, along with 1–2 “twin” pills, pills which differed only by one characteristic (color, size, shape). The PIT score was calculated as the sum of misidentifications weighted according to the degree of resemblance of the pills (0.5 for the twin, 1 for other or omission). Mistakes on how many pills per day resulted in +1. The patient’s knowledge of ARV treatment was considered satisfactory if the PIT score was lower than 1. The scoring system followed previously published norms [18]. MPR measures the amount of time an individual is in possession of their ARV pills as a proportion of the time between 2 ARV pick-ups [26]. Data for MPR were abstracted from the Electronic Dispensing Tool (EDT), a standardized pharmacy record system used to dispense ART at all public ART sites in Namibia, collecting data including date of ART pick-up, ART regimen, and number of pills dispensed. MPR was calculated for the entire time the patient was on ART until the viral load with the formula: number of days ARV dispensed/number of days between first and last ARV pick-up.

The standardized questionnaire was administered via face-to-face interviews by a trained interviewer. The questionnaire was translated into Oshiwambo and Afrikaans and back-translated and pre-tested to ensure accuracy. The questionnaire was administered in English, Oshiwambo or Afrikaans depending on the participant’s preference. English is the official language in Namibia; and Oshiwambo and Afrikaans are spoken in 37% and 24% of households respectively [27].

### Statistical Analysis

Univariate statistics were obtained for each variable included in this analysis. Frequencies and distributions were carefully examined for unusual values. For the outcome, we chose to look at viral load level at two thresholds, <1000 copies/mL and <5000 copies/mL. These two thresholds were chosen to reflect the World Health Organization (WHO) criteria for prevention of HIV drug resistance (1000 copies/mL) [28] and treatment failure (5000 copies/mL) [29]. MPR was analyzed as a continuous variable and then dichotomized at a threshold of 75%. The Likert item was dichotomized at two different thresholds for adherence (Likert A = Excellent, very good vs. good, fair, poor; Likert B = Excellent vs. very good, good, fair, poor). VAS was analyzed as a continuous variable and then dichotomized with adherent being defined as >95%. A satisfactory PIT score was <1. Self-reported 48-hour treatment interruptions were dichotomized at ≥1 treatment interruption over the entire period on ART.

Separate chi square analyses were conducted for each adherence measure to determine if associations existed between adherence and each viral load outcome. Bivariate associations were examined between other relevant demographic and potentially confounding variables and viral load using chi square tests for categorical variables and Wilcoxon Rank Sum tests for ordinal and continuous measures.

To assess bivariate associations between outcome variables and each of the adherence measures and demographic variables of interest, three statistical tests were used: Spearman rank correlation was used to measure the association between two continuous variables; Wilcoxon Rank Sum tests for the association between binary and continuous variables; and Kruskal-Wallis tests for the association between categorical and continuous variables.

The degree that adherence measures agreed with each other was assessed. Agreement among the dichotomized measures of adherence (VAS, Likert, PIT score and MPR, and self-reported treatment interruption) was assessed using Cohen’s kappa statistic.

For all statistical analyses, an alpha of 0.05 was used to evaluate statistically significant differences or associations. All analyses were performed using PASW version 18 (IBM; Armonk, New York).

## Results

Adherence to ART was assessed in 236 patients (Table 1), mean age 37.3 years. Patients were 54% female (53.6% of patients on ART in Windhoek are female) [unpublished data] and 86% single. Mean time on ART was 263 days. At time of ART initiation, 26% had WHO clinical stage 3 or 4; 9% had WHO clinical stage 3 or 4 at the time of the adherence assessment. Median CD4 cell count at ART initiation was 186 cells/mL. The mean weight at ART initiation was 58.5 kg and 61.0 kg at the time of the adherence assessment. ART starting regimens are listed in Table 1. The majority of patients (93%) reported no previous ART experience and a treatment supporter (78%). Median monthly income was 1000 Namibian Dollars (US$ 124) (mean monthly income for Namibia US$ 444) [30], and median distance from the ART clinic was 4 km. Forty percent had less than secondary school education and 59% had secondary school education.

**Table 1:** Characteristics of participants: 236 recent ART starters by viral load cutoff.

Characteristic	Overall	Viral load cutoff = 1000 c/mL(a)			Viral load cutoff = 5000 copies c/mL(a)		
		≥1000	<1000	P-value	≥5000	<5000	P-value
	(N = 236)	(n = 57)	(n = 179)		(n = 28)	(n = 208)	
	*Mean (SD),* *Median (Q1,* *Q3), or N (%)*	*Mean (SD),* *Median (Q1,* *Q3), or N (%)*	*Mean (SD),* *Median (Q1,* *Q3), or N (%)*		*Mean (SD),* *Median (Q1,* *Q3), or N (%)*	*Mean (SD),* *Median (Q1,* *Q3), or N (%)*	
Age	37.3 (8.5)	38.0 (8.4)	37.1 (8.6)	0.480	38.1 (8.5)	37.2 (8.5)	0.614
Sex							
Male	108 (45.8)	27 (47.4)	81 (45.3)	0.780	10 (35.7)	98 (47.1)	0.256
Female	128 (54.2)	30 (52.6)	98 (54.7)		18 (64.3)	110 (52.9)	
Marital status							
Single	202 (85.6)	50 (87.7)	152 (84.9)	0.681	24 (85.7)	178 (85.6)	0.869
Married	32 (13.6)	7 (12.3)	25 (14.0)		4 (14.3)	28 (13.5)	
Widowed	2 (0.8)	0 (0)	2 (1.1)		0 (0)	2 (1.0)	
Education							
None	31 (13.1)	9 (15.8)	22 (12.3)	0.225	2 (7.1)	29 (13.9)	0.507
Primary	63 (26.7)	18 (31.6)	45 (25.1)		11 (39.3)	52 (25.0)	
Secondary	139 (58.9)	29 (50.9)	110 (61.5)		15 (53.6)	124 (59.6)	
Diploma	2 (0.8)	0 (0)	2 (1.1)		0 (0)	2 (1.0)	
Bachelor’s degree	1 (0.4)	1 (1.8)	0 (0)		0 (0)	1 (0.5)	
Monthly income (Namibian $)	1000 (350, 1800)	1000 (350, 1900)	1000 (325, 1800)	0.867	800 (219, 1975)	1000 (350, 1788)	0.641
CD4 at start (cells/mL)	186 (124, 227)	163 (89, 209)	195 (137, 232)	0.016	158 (87, 236)	189 (134, 226)	0.294
Starting ART regimen^(b)(c)^							
AZT/3TC/EFV	21 (8.9)	4 (7.0)	17 (9.5)	0.498	3 (10.7)	18 (8.7)	0.832
AZT/3TC/NVP	130 (55.1)	34 (59.6)	96 (53.6)		14 (50.0)	116 (55.8)	
D4T/3TC/NVP	15 (6.4)	4 (7.0)	11 (6.1)		3 (10.7)	12 (5.8)	
TDF/3TC/EFV	33 (14.0)	7 (12.3)	26 (14.5)		5 (17.9)	28 (13.5)	
TDF/3TC/NVP	36 (15.3)	7 (12.3)	29 (16.2)		3 (10.7)	33 (15.9)	
D4T/3TC/LVP/r	1 (0.4)	1 (1.8)	0 (0)		0 (0)	1 (0.5)	
Days on ART	263.1 (57.0)	255.7 (56.0)	265.4 (57.4)	0.263	250.0 (54.2)	264.8 (57.3)	0.196
Prior ART exposure							
Never on ART	219 (92.8)	53 (94.6)	166 (93.3)	0.830	24 (88.9)	195 (94.2)	0.462
Transfer in on ART	14 (5.9)	3 (5.4)	11 (6.2)		3 (11.1)	11 (5.3)	
PMTCT	1 (0.4)	0 (0)	1 (0.6)		0 (0)	1 (0.5)	
WHO clinical stage at start(d)							
1	119 (50.4)	27 (49.1)	92 (53.2)	0.860	14 (51.9)	105 (52.2)	0.869
2	48 (20.3)	15 (27.3)	33 (19.1)		6 (22.2)	42 (20.9)	
3	50 (21.2)	10 (18.2)	40 (23.1)		4 (14.8)	46 (22.9)	
4	11 (4.7)	3 (5.5)	8 (4.6)		3 (11.1)	8 (4.0)	
WHO clinical stage at end							
1	191 (83.0	38 (67.9)	153 (87.9)	<0.001	16 (59.3)	175 (86.2)	0.001
2	19 (8.3)	7 (12.5)	12 (6.9)		6 (22.2)	13 (6.4)	
3	15 (6.5)	10 (17.9)	5 (2.9)		5 (18.5)	10 (4.9)	
4	5 (2.2)	1 (1.8)	4 (2.3)		0 (0)	5 (2.5)	
Weight at start (kg)	58.5 (10.4)	58.6 (11.1)	58.5 (10.2)	0.933	59.0 (12.4)	58.4 (10.2)	0.787
Weight at end (kg)	61.0 (10.9)	61.0 (11.0)	61.0 (11.0)	0.973	61.9 (13.4)	60.9 (10.6)	0.672
Use of cotrimoxazole							
Never	7 (3.0)	1 (1.8)	6 (3.4)	0.594	0 (0)	7 (3.4)	0.540
Current	223 (94.5)	55 (98.2)	168 (95.5)		27 (100)	196 (95.6)	
Stopped	2 (0.8)	0 (0)	2 (1.1)		0 (0)	2 (1.0)	
Use of treatment supporter							
Yes	184 (78.0)	44 (77.2)	140 (78.2)	0.872	20 (71.4)	164 (78.8)	0.374
No	52 (22.0)	13 (22.8)	39 (21.8)		8 (28.6)	44 (21.2)	
Distance travel to clinic (km)	4.0 (2.0, 6.0)	5.0 (3.0, 6.0)	3.0 (2.0, 6.0)	0.313	5.0 (3.0, 6.0)	3.0 (2.0, 6.0)	0.340

(a) c/mL = copies per milliliter.

(b) ART = antiretroviral therapy.

(c) NVP = nevirapine; EFV = efavirenz; TDF = tenofovir; 3TC = lamivudine; AZT = zidovudine; D4T = stavudine; LVP/r = lopinavir/ritonavir.

(d) WHO = World Health Organization.

Median and mean adherence was 100% and 98.1% respectively by 30-day VAS. Ninety-six percent of respondents had a VAS percent of >95%. By the 30-day Likert item 85% reported “excellent” or “very good” adherence and 48% of respondents reported “excellent” adherence. Ninety-one percent of respondents had a satisfactory PIT score. Median and mean adherence by MPR was 100.0% and 98.8%. Ninety-three percent had ≥75% MPR. Only 3% of respondents reported having one or more treatment interruption of ≥48 hours. (Table 2).

**Table 2:** Adherence measures by viral load cutoff.

AdherenceMeasure	Overall	Viral loadcutoff = 1000 c/mL(a)			Virologic Failure	Viral loadcutoff = 5000 c/mL(a)			VirologicFailure
	(N = 236)	≥1000(n = 57)	<1000(n = 179)	P-value	Odds Ratio(95% CI)	≥5000(n = 28)	<5000(n = 208)	P-value	Odds Ratio(95% CI)
	N (%)	N (%)	N (%)			N (%)	N (%)		
MPR(b) (≥75%)									
Adherent	220 (93.2)	50 (87.7)	170 (95.0)			23 (82.1)	197 (94.7)		
Non-adherent	16 (6.8)	7 (12.3)	9 (5.0)	0.058	2.64 (0.94, 7.46)	5 (17.9)	11 (5.3)	0.013	3.89 (1.24, 12.21)
Likert A(c)									
Adherent	200 (84.7)	50 (87.7)	150 (83.8)			23 (82.1)	177 (85.1)		
Non-adherent	36 (15.3)	7 (12.3)	29 (16.2)	0.473	0.72 (0.30, 1.76)	5 (17.9)	31 (14.9)	0.683	1.24 (0.44, 3.52)
Likert B(d)									
Adherent	113 (47.9)	27 (47.4)	86 (48.0)			15 (53.6)	98 (47.1)		
Non-adherent	123 (52.1)	30 (52.6)	93 (52.0)	0.929	1.03 (0.57, 1.87)	13 (46.4)	110 (52.9)	0.521	0.77 (0.35, 1.70)
VAS(e) (>95%)									
Adherent	226 (95.8)	55 (96.5)	171 (95.5)			27 (96.4)	199 (95.7)		
Non-adherent	10 (4.2)	2 (3.5)	8 (4.5)	0.754	0.78 (0.16, 3.77)	1 (3.6)	9 (4.3)	0.852	0.82 (0.10, 6.72)
PIT(f) score (<1)									
Adherent	213 (90.6)	53 (93.0)	160 (89.9)			25 (89.3)	188 (90.8)		
Non-adherent	22 (9.4)	18 (10.1)	4 (7.0)	0.485	0.67 (0.22, 2.07)	19 (9.2)	3 (10.7)	0.793	1.19 (0.33, 4.29)
Treatment Int(g)									
Adherent	229 (97.0)	54 (94.7)	175 (97.8)			27 (96.4)	202 (97.1)		
Non-adherent	7(3.0)	3 (5.3)	4 (2.2)	0.364(h)	2.43 (0.53, 11.20)	1 (3.6)	6 (2.9)	0.592(h)	1.25 (0.14, 10.75)

(a) c/mL = copies per milliliter.

(b) MPR = Medication possession ratio.

(c) Likert A = Excellent, very good vs good, fair, poor.

(d) Likert B = Excellent vs very good, good, fair, poor.

(e) VAS = Visual analogue scale.

(f) PIT = Pills identification test.

(g) Treat Int = Self-reported treatment interruptions ≥1.

(h) Fisher’s exact test.

Agreement between adherence measures was poor with low Kappa statistic values. The VAS had higher agreement with self-reported treatment interruptions (Kappa = 0.27; p<0.001 for ≥1 treatment interruptions) than other clinic-based measures. The second highest agreement was VAS with 30-day Likert (Kappa = 0.21; p<0.001 for Likert item excellent and very good vs. good, fair and poor).

HIV-1 RNA was determined 6 months after ART initiation; 179/236 (76%) had HIV-1 RNA <1000 copies/mL, and 208/236 (88%) had HIV-1 RNA <5000 copies/mL. MPR (continuous variable) was significantly associated with viral suppression at <5000 copies/mL (p = 0.036). Having MPR <75% was significantly associated with virologic failure at ≥5000 copies/mL with OR 3.89 (1.24, 12.21), p = 0.013. MPR <75% had borderline significant association virologic failure at ≥1000 copies/mL with OR 2.64 (0.94, 7.46), p = 0.058. No other adherence measure had a significant association with viral load at ≥5000 copies/mL or ≥1000 copies/mL.

## Discussion

This study is the first reported ART adherence assessment among HIV-infected patients in Namibia. The level of adherence observed in the three public ART delivery sites in Windhoek, Namibia was high as estimated by all five adherence measures. This finding is consistent with previously published levels of reported adherence in other African settings [16], [31]–[33].

We used five different measures of adherence in this study to assess for a cross-cultural and simple measure of adherence associated with short-term virologic response. Viral load 6 months after ART initiation was used as a marker for short-term virologic response and was tested for associations with the different adherence measures. As shown previously in other settings [6], [13], [21], [26], in Namibia we confirm that MPR was associated with short-term virologic response. The threshold of <75% MPR was significantly associated with virologic failure ≥5000 copies/mL at 6 months. This finding suggests that MPR may be a useful tool to help identify patients at risk for early virologic failure in Namibia and similar settings. Unlike patient self-reported adherence measures, which are prone to recall or social desirability bias, MPR is an objective measure because it does not rely on asking the patient, but instead uses routine data from pharmacy visits. Additionally, the MPR captures treatment interruptions because it takes into account time periods without medication coverage. Therefore, MPR may be an important tool in resource-limited settings where many patients may experience treatment interruption due to lack of access to medications.

No other measure of adherence in this study was found to be significantly associated with short-term virologic response. In addition, agreement between adherence measures was poor. These results could be explained by a variety of cultural-linguistic factors and/or limitations with the tools themselves such as recall and social desirability bias. In addition, some tools may be limited in their adherence assessment depending on the reasons for poor adherence and pattern of missed doses as detailed below.

The level of adherence by 30-day Likert in our study was high, but was not associated with virologic suppression. Although, the 30-day Likert item has been validated in other settings, the overestimation of adherence by these types of self-reported adherence tools has been reported [34]. The discrepancy between relatively high self-reported levels of adherence and low levels of virologic suppression at 6 months suggests that there may have been social pressure to report optimal adherence in this population. It is worth mentioning that the Likert item only assessed perceived adherence and not actual adherence by asking, “How well do you think you took your ARVs in the past 30 days?” Therefore, a respondent experiencing treatment interruptions due to lack of access to their ART clinic may still report excellent adherence by Likert because he/she did not choose to be non-adherent. The same could be said of VAS. In our study, VAS seemed to have performed even worse than the 30-day Likert item. The overwhelming number of VAS responses was 100% adherence (226 of 236; 95.8%). Additionally, seven of the ten not responding 100% adherence were 50%. We hypothesize that in this setting the concept of percentages used in the VAS may not be understood as well as discrete categories used in the Likert item. Self-reported treatment interruptions was also not associated with virologic failure. An overwhelming proportion of respondents reported never having a 48-hour treatment interruption (97%). The literature has demonstrated that ≥48 hours of unplanned treatment interruptions are associated with the development of HIV drug resistance and increased risk of treatment failure [35]–[36]. Although information about treatment interruptions is vital to predicting treatment failure, self-reported treatment interruptions may not be a valid method of obtaining this information. Self-reported treatment interruptions may have been affected by recall bias or social desirability bias. Also, treatment interruptions may have been interpreted to be self-imposed interruptions of therapy instead of interruptions due to lack of access to medications.

To our knowledge, this is the first study assessing PIT in Sub-Saharan Africa. As the PIT requires minimal use of words because it involves primarily identification of the pills or pictures of pills [18], it has been suggested that PIT may be useful for assessing adherence in resource-limited settings and may be a better cross-cultural tool. However, we found no association of the PIT with viral suppression. Importantly, a large proportion of patients correctly identified their ARV medication (91%). In the original context in France the PIT was developed and validated at a time when ART regimens were more complex. In settings where ART regimens are simplified and fewer regimens are in use such as in resource-limited settings, the PIT may not discriminate between high and low adherence. Also in the original context where the PIT was developed and assessed [18], non-adherence was mainly related to “perceived side effects”, carelessness and forgetting to take medications (individual patient adherence) and not associated with structural barriers (free drugs, home-hospital travel reimbursements, drug continuity). In contrast, treatment interruptions due to structural barriers have been demonstrated to be a critical reason for missed doses in resource-limited setting [36]. Therefore, we hypothesize that many subjects in our cohort recognized and identified their pills perfectly, but simply experienced treatment interruptions due to structural barriers, and thus developed HIV drug resistance and/or treatment failure. In this case where structural barriers may be more important than individual patient adherence, MPR would best capture this partial exposure to ARV drugs.

This study has several limitations. First, we conducted the adherence assessment in Windhoek, which is not representative of the country. However, because Windhoek has a mixture of the different socio-economic and ethnic populations present in Namibia, we would expect similar understanding and usefulness of these adherence tools. Results though may not be as generalizable to rural populations. Second, adherence assessments were conducted a mean two months after the 6-month viral load. This lag period leaves open the possibility that the lack of association between self-reported adherence and viral load was due to a change in viral load [37] or patient adherence during that time. Third, the 6-month viral load may not be the best surrogate for long-term virologic response. However, recent data indicate that the 6-month viral load may predict subsequent survival, retention in care and switch to second-line therapy [38]. Importantly, identification of an adherence measure that is associated with 6-month viral load may be useful for early identification of those who are at risk of suboptimal adherence. These at-risk patients can then receive targeted interventions to optimize patient care and minimize the emergence of drug resistance.

In conclusion, this study provides the first data from Namibia describing levels of ARV adherence comparing multiple adherence measures. The levels of adherence were high with all adherence measures, but self-reported adherence measures were not associated with virologic failure. Depending on the reasons and patterns of missed doses in particular populations, some tools may be more helpful than others. In resource-limited settings, self-reported adherence measures may be less useful tools because non-adherence and thus virologic failure may be due more to lack of access to medications rather than individual reasons for poor adherence. MPR was found to be associated with short-term virologic response, which suggests its utility in early identification of patients at high risk for virologic failure in resource-limited settings. Future research should focus on identifying optimal adherence measure tools for resource-limited settings where lack of access to medications may be the primary driver for treatment failure.
